# Chitosan-Based Ciprofloxacin Extended Release Systems: Combined Synthetic and Pharmacological (In Vitro and In Vivo) Studies

**DOI:** 10.3390/molecules27248865

**Published:** 2022-12-13

**Authors:** Anton R. Egorov, Aleh V. Kurliuk, Vasili V. Rubanik, Anatoly A. Kirichuk, Omar Khubiev, Roman Golubev, Nikolai N. Lobanov, Alexander G. Tskhovrebov, Andreii S. Kritchenkov

**Affiliations:** 1 Faculty of Science, Peoples’ Friendship University of Russia (RUDN University), Miklukho-Maklaya St. 6, Moscow 117198, Russia; 2 Department of General and Clinical Pharmacology, Vitebsk State Medical University, Frunze Av. 27, 210009 Vitebsk, Belarus; 3 Metal Physics Laboratory, Institute of Technical Acoustics NAS of Belarus, Ludnikova Prosp. 13, 210009 Vitebsk, Belarus

**Keywords:** chitosan, ciprofloxacin, antibacterial activity

## Abstract

Ciprofloxacin is one of the most effective antibiotics, but it is characterized by a range of side effects. Elaboration of drug-releasing systems which allow to diminish toxicity of ciprofloxacin is a challenging task in medicinal chemistry. The current study is focused on development of new ciprofloxacin releasing systems (CRS). We found that ultrasound efficiently promotes *N,N′*-dicyclohexyl carbodiimide-mediated coupling between COOH and NH_2_ functionalities in water. This was used for conjugation of ciprofloxacin to chitosan. The obtained ciprofloxacin/chitosan conjugates are capable of forming their self-assembled nanoparticles (SANPs) in aqueous medium. The SANPs can be additionally loaded by ciprofloxacin to form new CRS. The CRS demonstrated high loading and encapsulation efficiency and they are characterized by extended release profile (20 h). The elaborated CRS were tested in vivo in rats. The in vivo antibacterial effect of the CRS exceeded that of the starting ciprofloxacin. Moreover, the in vivo acute and subacute toxicity of the nanoparticles was almost identical to that of the chitosan, which is considered as the non-toxic biopolymer.

## 1. Introduction

Infectious diseases currently are a major problem and occupy a significant part in the causes of death of the human population [1]. Etiotropic therapy of bacterial infectious diseases is aimed mainly at killing of infectious agents. Among a great variety of antibacterial agents, fluoroquinolone antibiotics are extremely effective. They are often the only medicament that can help in saving a patients’ life [2]. An important problem in the use of fluoroquinolones, like other antibiotics, is their systemic toxicity [3] and a rather rapid elimination [4,5]. These problems can be solved by drug conjugation to a polymer matrix or encapsulation in micro- or nanoparticles [6,7,8]. In addition, we have previously shown that the conjugation of an antibacterial compound to a polymer matrix can significantly diminish its toxicity without reducing the antibacterial effect [9].

Among various polymers available for the conjugation of antibacterial drugs, polysaccharides occupy a special place, since they are characterized by biocompatibility, biodegradability, lack of carcinogenicity, immunogenicity and allergenic properties [10]. Among polysaccharides, chitosan has the greatest advantages, because it contains a primary amino group, which provides preparative convenience and ease of chemical modification [11]. In addition, chitosan itself possesses an antibacterial effect [12,13]. Moreover, chitosan is regarded as one of the cheapest natural polymers, and the most abundant biopolymer after cellulose [12]. Undoubtedly, chitosan can be attributed to inexhaustible resources [14]. These outstanding advantages of chitosan made it an attractive and promising object for elaboration of various systems for targeted delivery and prolonged release of drugs [15]. The impressive advances in the development of these systems based on chitosan are carefully reviewed in recent papers, reviews and books [16,17,18,19,20].

To the best of our knowledge, fluoroquinolone-loaded nanoparticles of chitosan or its derivatives, obtained only by ionic gelation, are described in the literature [21,22,23]. Conjugates of fluoroquinolones with chitosan are not described. In addition, the literature does not describe amphiphilic self-assembled nanoparticles loaded with fluoroquinolone, and such systems are also of undoubted interest as targeted drug delivery and controlled release systems.

**Scheme 1:**
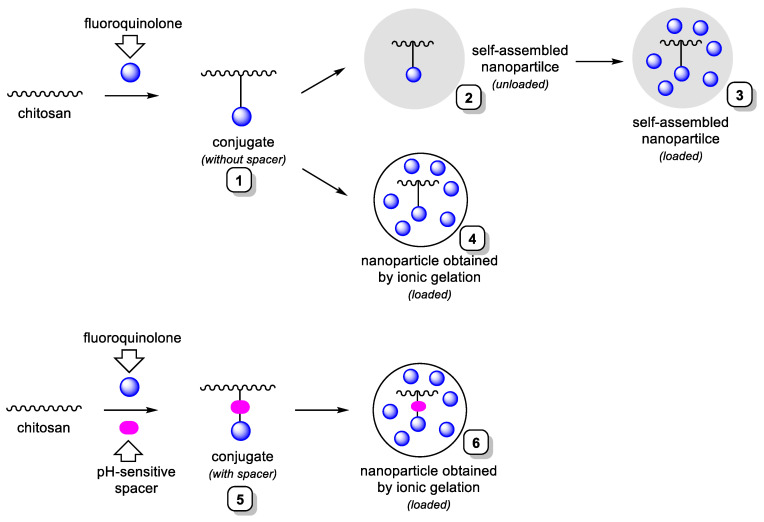
General strategy of preparation of the fluoroquinolone-based antibacterial systems.

Within the frames of this work, we hypothesized that the introduction of a sufficiently hydrophobic fluoroquinolone (i.e., ciprofloxacin) into the polymer chain of chitosan can lead to formation of fluoroquinolone conjugates (**1**, see Scheme 1), capable of self-assembly into amphiphilic nanoparticles (**2**, see Scheme 1); and these nanoparticles **2** (see Scheme 1) can be additionally loaded with fluoroquinolone (**3**, see Scheme 1). Conjugates of type (**1**, see Scheme 1), which are not capable of self-assembly, can be converted into nanoparticles loaded with fluoroquinolone (**4**, see Scheme 1) by ionic gelation. In addition, we attempted to prepare fluoroquinolone conjugates in which fluoroquinolone is bound to chitosan through a pH-sensitive spacer (**5**, see Scheme 1), suggesting that such systems are capable of releasing fluoroquinolone at slightly acidified pH values characteristic to the inflammatory process of bacterial etiology [24]. In addition, conjugates with a spacer (**5**, see Scheme 1) can also be further converted into fluoroquinolone-loaded nanoparticles by ionic gelation (**6**, see Scheme 1).

Based on the release profile of the prepared systems, we planned to identify the leading fluoroquinolone-based system and study its antibacterial effect both in vitro and in vivo. It should be noted that the systems based on fluoroquinolones and chitosan described in the literature have only been studied in vitro. The results of the elaboration of the proposed hypothesis are presented in the sections that follow below.

## 2. Results and Discussion

### 2.1. Synthesis of Ciprofloxacin-Chitosan Conjugates in Which Ciprofloxacin Is Directly Attached to the Polymer Chain (Scheme 1, System 1)

In this work, ciprofloxacin was chosen because this antibiotic is (i) one of the most effective even in the case of severe septic conditions, (ii) but, at the same time, quite toxic and is characterized by a number of dangerous side effects [25]. Therefore, the creation of systems for the prolonged release of ciprofloxacin, which can reduce its toxic effect, is an important task of medicinal chemistry and pharmacology [26]. Ciprofloxacin molecule due to its carboxylic function –COOH can be conjugated with primary amino group of chitosan –NH_2_. For amide bound formation through conjugation of carboxylic and primary amine groups, conventional carbodiimide method is used [27]. This method involves widely available and cheap carbodiimide DCC (*N,N′-*dicyclohexylcarbodiimide). However, insolubility of DCC in aqueous media strongly limits its application in chitosan chemistry. Since the main solvent for chitosan is water (at acidic pH values), for conjugation of chitosan with carboxylic acids, water soluble carbodiimide 1-ethyl-3-(3-dimethylaminopropyl)carbodiimide) (EDC) is preferred [27]. The application of EDC results in significantly higher conversion of amino groups to the corresponding amide bonds, since the reaction proceeds in a homogeneous regime. However, it should be noted that EDC is much more expensive than DCC.

Recently, we reported that ultrasonic irradiation can promote a number of heterogeneous organic transformation of chitin and chitosan resulting in dramatic decrease of the reaction time and the required amount of reagent, wherein it leads to increase in the degree of conversion (in comparison with the conventional, i.e., ultrasound-free conditions) [28,29,30,31]. Moreover, it is often possible to find such acoustic conditions that promote the desired reaction without causing ultrasonic degradation of chitosan backbone [9,32,33]. As an extension of our previous findings, we attempted to promote the DCC-mediated conjugation of ciprofloxacin with chitosan using ultrasound.

**Scheme 2:**
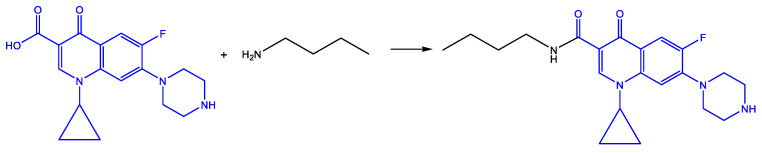
Model DCC-mediated reaction.

Firstly, we optimized acoustic conditions for the model DCC-mediated reaction of ciprofloxacin with *n-*butylamine in water (Scheme 2).

We found that the optimal conditions for the model reaction lie in the range of frequencies 90–110 kHz and output powers 270–290 W. At these conditions the reaction finishes in 20 min (TLC monitoring) giving the desired amide in 90% preparative yield. Application of harder acoustic conditions makes the reaction not selective (nine new spots on TLC). At lower frequency and power values, the reaction proceeds slowly and results in lower preparative yields of the product.

**Scheme 3:**
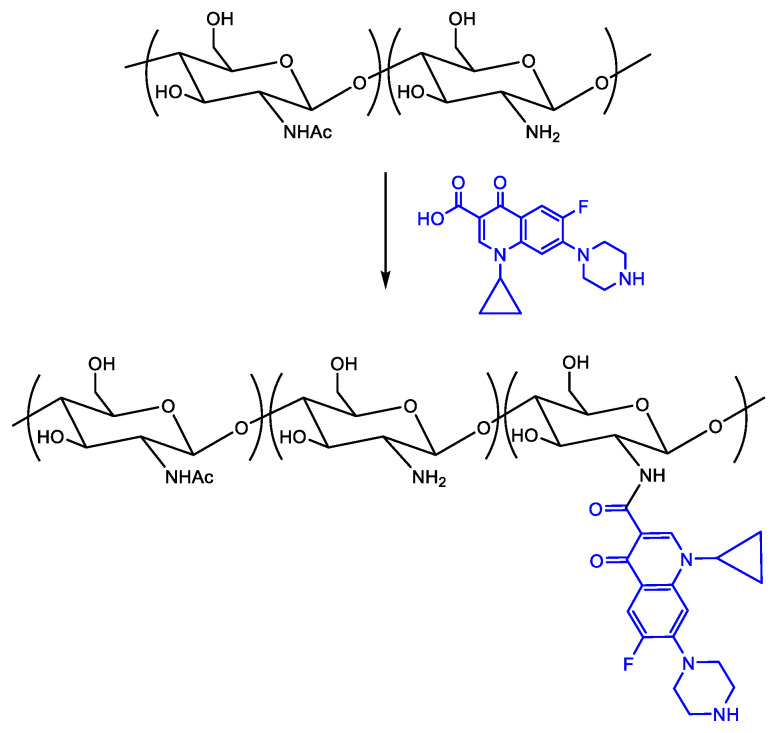
Conjugation of ciprofloxacin with chitosan.

In our previous works, we demonstrated that the ultrasonic treatment of chitosan at 80–100 kHz and 250–300 W during ca. 15–20 min does not provoke any depolymerization of the chitosan backbone (see [9] and references in it). Thus, transferring the conditions of the model reaction to the reaction with the participation of chitosan, we decided to use the frequency 100 kHz and the power 280 W as acoustic parameters. The DCC-mediated reaction of chitosan with ciprofloxacin (Scheme 3) was studied under both ultrasound-free and ultrasonic conditions.

We estimated the effect of the required excess of ciprofloxacin to reach the same degree of substitution (0.05, 0.10, and 0.20) with and without influence of ultrasonic irradiation. We treated reaction mixtures of ciprofloxacin and chitosan at pH = 3, T = 50 °C during 5 h (without ultrasound) or 20 min (with ultrasonic irradiation at 100 kHz, 280 W). The ultrasound-free reaction at a molar ratio chitosan/ciprofloxacin 1:0.5 or 1:1.5 furnished *N-*substituted conjugates with the degrees of substitution of 0.05 or 0.10, respectively. When the molar ration chitosan/ciprofloxacin was increased to 1:4, the reaction resulted in the formation of higher substituted products (degree of substitution 0.20). Application of ultrasonic approach dramatically diminished both the reaction time and required excess of ciprofloxacin, subject to the achievement of the same degree of substitution. These results are presented in Table 1. They can be explained by the mechanical activation of macromolecular coils, as well as the formation of active species such as radicals [34]. In addition, the collapse of cavitation bubbles is accompanied by the dissipation of a large amount of energy, which, in turn, results in an increase in the reaction rate [35]. It is also important to note that the ultrasonic parameters used make it possible to preserve the integrity of the polysaccharide chain. This means that the characteristics of the final material, which directly depend on the molecular weight and the degree of deacetylation of the polymer, do not change.

**Figure 1:**
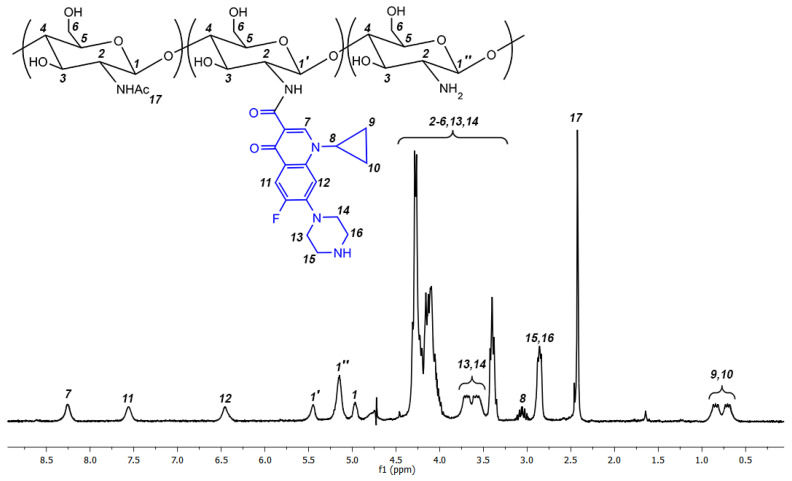
The typical ^1^H NMR spectrum of ciprofloxacin/chitosan conjugate.

The resultant ciprofloxacin/chitosan conjugates were characterized by ^1^H NMR spectroscopy. The typical ^1^H NMR spectrum with signal assessment is presented in Figure 1. Degree of substitution (DS) of the conjugates was calculated according to the formula: DS = I(***1***′), while I(***1***) + I(***1***′) + I(***1***″) = 1. The code names of the synthesized polymers, their degree of substitution and molecular weights of the starting chitosan are presented in Table 2. The abbreviated names of the products (see Table 2) should be decoded as follows: **C-CS-I-L**: **C**—ciproloxacin-conjugated derivative, **CS**—chitosan, **I**—degree of substitution 0.05, **L**—low molecular weight (3.5 × 10^4^, see Section 2.1).

The resultant polymers are insoluble in water, but soluble in 1% acetic acid or 1% HCl solution. Moreover, being dissolved in 1% acetic acid or 1% HCl, the polymers do not precipitate from the solution, if its pH is adjusted to 7.0 by addition of sodium hydrocarbonate.

**Figure 2:**
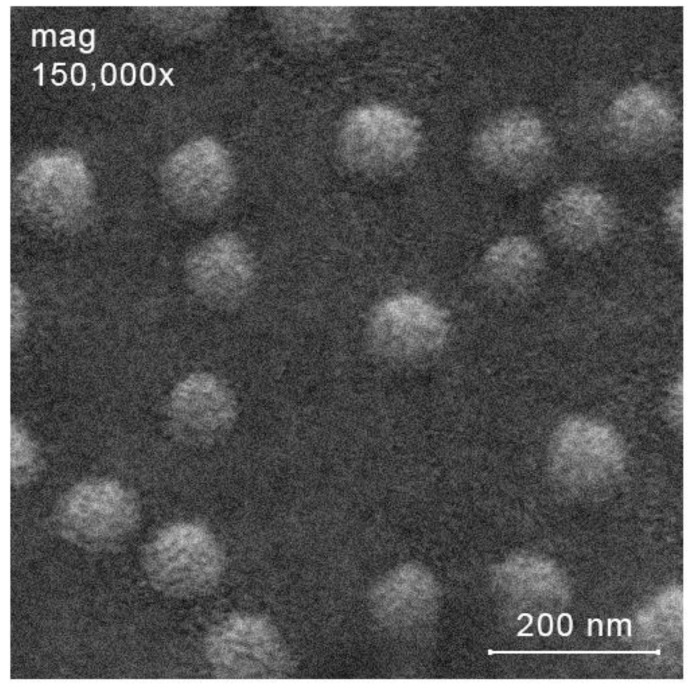
SEM image of self-assembled nanoparticles (**NPs-C-CS-III-M**).

At pH = 7.0, **C-CS-I-M**, **C-CS-II-M** and **C-CS-III-M** self-organize into the corresponding nanoparticles after sonication. Optimization of acoustic parameters revealed, that at 5 min of ultrasonic treatment with frequency 30 kHz and output power 300 W, the formed nanoparticles are characterized by unimodal size distribution. The characteristics of the resultant nanoparticles (Scheme 1, system 2) are presented in Table 3. The hydrodynamic diameter of the self-assembled nanoparticles has strong dependency on the degree of substitution of the starting chitosan/ciprofloxacin conjugate. More substituted polymers self-assemble into nanoparticles with larger hydrodynamic diameter. The ζ-potential of the nanoparticles formed also increases with the increase in degree of substitution of the starting conjugates, but in significantly less extent. All nanoparticles are redispersible after lyophilization. Scanning electron microscopy of the resultant nanoparticles confirms their size values obtained by dynamic light scattering and also demonstrates the spherical shape of the nanoparticles (see, for example, Figure 2).

### 2.2. Synthesis of Ciprofloxacin-Chitosan Conjugates in Which Ciprofloxacin Is Linked to the Polymer Chain through a pH-Sensitive Linker (Scheme 1, System 5)

**Scheme 4:**
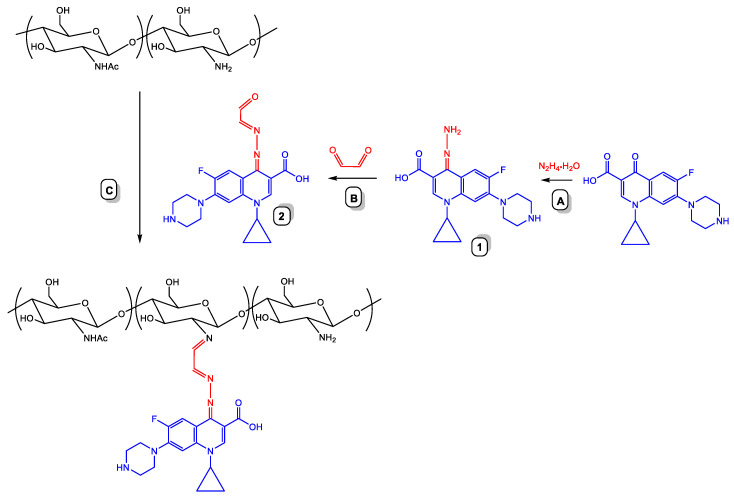
Conjugation of ciprofloxacin through pH sensitive linker with chitosan.

In many instances, conjugation of pharmacologically active compound with polymer matrix through pH-sensitive linker results in dramatic improve of the drug release profile. In this study, we prepared ciprofloxacin chitosan derivatives, in which ciprofloxacin is conjugated with the polysaccharide backbone through the pH sensitive hydrazone linker based on glyoxsal. The conjugation strategy via the linker is presented in Scheme 4. Ciprofloxacin has been involved in the *Ad_N_-E* reaction with hydrazine to form the corresponding hydrazone **1** (Scheme 4, A). Hydrazone **1** interacted with glyoxal to give rise to **2** (Scheme 4, B). Substance **2,** bearing an aldehyde group, reacted with the free amine groups of the chitosan furnishing the corresponding Schiff base (Scheme 4, C).

Compound **1** is easily formed in almost quantitative yield. At the same time, the synthesis of compound **2** is associated with some preparative difficulties. After optimising the synthesis conditions, we found that the highest isolated yield (ca. 70%) of product **2** were observed when excess of 3.5 equivalents of glyoxal was used. Purification of product **2** requires column chromatography.

Interaction of **2** with chitosan proceeds much slower that with conventional aldehydes such as acetaldehyde or benzaldehyde. Apparently, this fact can be explained by the influence of an open heterochain system of conjugated double bonds in the molecule of compound **2**, covering significantly more than 10 atoms, including the atoms of the aldehyde group. Interaction with chitosan at the first stage of the reaction leads to the destruction of the conjugated system in the region of the carbonyl group. Partial destruction of the polyconjugated system is disadvantageous, which causes reduced reactivity of compound **2**.

Recently, we reported that ultrasonic irradiation promotes Schiff base formation arising from interaction of aromatic or aliphatic aldehydes with chitosan. Acoustic conditions to promote Schiff base formation are extremely wide, however, those that make it possible to promote this reaction without side rapture of the chitosan backbone lie in the region 80 kHz 250 W. In the current work, we applied these conditions to the reaction of chitosan with compound **2** and estimated the effect of the ultrasound with the effect of ultrasound-free conditions (Table 4). We optimized both ultrasonic and ultrasound-free conditions to prepare chitosan derivatives with the same degree of substitution that were obtained in Section 3.1. The data provided in Table 4 clearly demonstrate that ultrasonic treatment of the reaction mixtures results in significant decrease of both, the excess of compound **2** and reaction time, to achieve the same degree of substitutions as those reached under ultrasound-free conditions.

The code names of the resultant polymers (Scheme 1, system 5), and their correspondence to their degree of substitution and to the molecular weights of the starting chitosans are presented in Table 5.

The resultant polymers with degree of substitution 0.11 and 0.22 (**C-SP-CS-II-L** and **C-SP-CS-III-L**) derived from low-molecular-weight chitosan (3.5 × 10^4^ Da) are water-soluble, while other conjugates are insoluble in water. All conjugates are soluble in 1% acetic acid or 1% hydrochloric acid. However, if the pH value of the acidic solution is adjusted to 7.0 by addition of sodium hydrocarbonate, the conjugates precipitate (expect water-soluble **C-SP-CS-II-L** and **C-SP-CS-III-L**). All prepared ciprofloxacin-conjugated chitosan derivatives with the pH sensitive spacer do not self-organize into nanoparticles.

**Figure 3:**
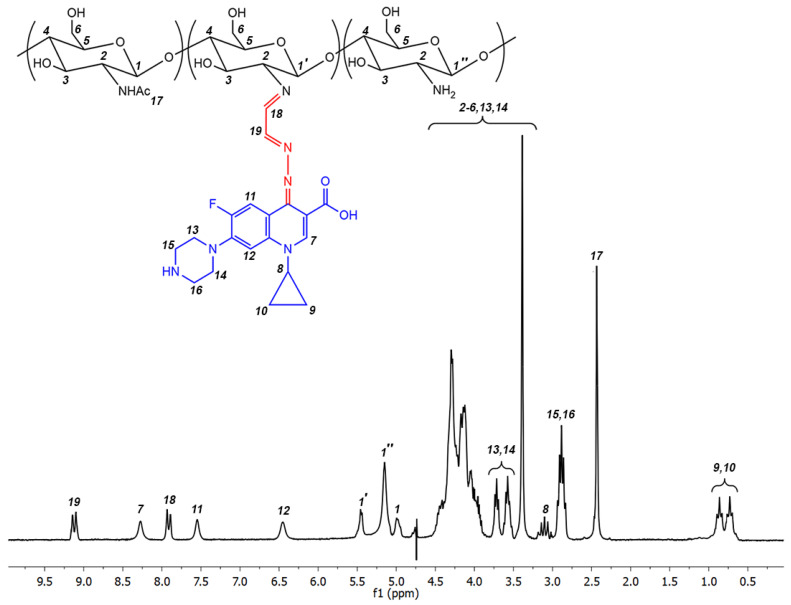
^1^H NMR spectrum of C-SP-CS-III-M.

The obtained chitosan derivatives were characterized by ^1^H NMR spectroscopy. The typical ^1^H NMR spectrum with the signal assessment is presented in Figure 3.

In the previous section, we prepared self-assembled nanoparticles of spacer-less ciprofloxacin conjugates with chitosan of medium molecular weight (7.1 × 10^4^ Da). In the current section, we demonstrated that spacer-containing ciprofloxacin conjugates with chitosan of medium molecular weight (7.1 × 10^4^ Da) do not spontaneously form nanoparticles. Consequently, we attempted to prepare the corresponding nanoparticles using the conventional ionic gelation method. Optimizing the ionic gelation conditions, we were able to synthesize the nanoparticles of **C-SP-CS-I-M**, **C-SP-CS-II-M** and **C-SP-CS-III-M** close in their size and ξ-potential to those prepared from **C-CS-I-M**, **C-CS-II-M** and **C-CS-III-M** in the previous section. As a gelating agent, we used sodium tripolyphosphate (TPP). The characteristics (hydrodynamic diameter and ξ-potential) of the obtained nanoparticles are presented in Table 6.

**Figure 4:**
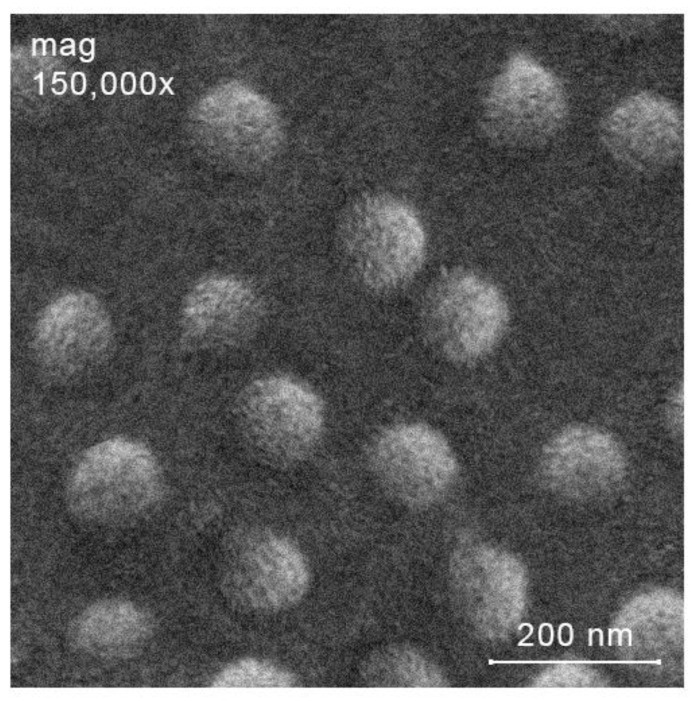
SEM image of the nanoparticles prepared by ionic gelation method (**NPs-C-SP-CS-III-M**).

The resultant nanoparticles are redispersible after lyophilization. Scanning electron microscopy of the synthesized nanoparticles confirms their hydrodynamic diameter values obtained by dynamic light scattering and also shows the nanoparticles are of spherical shape (see, for example, Figure 4).

### 2.3. Preparation of Ciprofloxacin-Loaded Nanoparticles

The ciprofloxacin-loaded nanoparticles were prepared by one of two suitable methods (i) loading of self-assembled nanoparticles (**NPs-C-CS-I-M**, **NPs-C-CS-II-M** and **NPs-C-CS-III-M**) derived from the corresponding polymers **C-CS-I-M**, **C-CS-II-M** and **C-CS-III-M**, or (ii) ionic gelation method for all other polymers, since other polymers do not self-organize into nanoparticles.

#### 2.3.1. Preparation of Ciprofloxacin-Loaded Nanoparticles from **NPs-C-CS-I-M**, **NPs-C-CS-II-M** and **NPs-C-CS-III-**M (Scheme 1, System 3)

Ciprofloxacin-loaded nanoparticles **C-L-NPs-C-CS-I-M**, **C-L-NPs-C-CS-II-M** and **C-L-NPs-C-CS-III-M** were prepared from the corresponding **NPs-C-CS-I-M**, **NPs-C-CS-II-M** and **NPs-C-CS-III-M** by their loading with ciprofloxacin under sonication. 1 mg of ciprofloxacin dissolved in ethanol was added to 5 mg of **NPs-C-CS-I-M**, **NPs-C-CS-II-M** or **NPs-C-CS-III-M** suspended in water. The fraction of loaded ciprofloxacin was estimated by the ciprofloxacin concentration in the supernatant after loading and removal of the loaded nanoparticles by centrifugation. Thus, we evaluated loading efficiencies, and these values were used to calculate the encapsulation efficiencies (i.e., the mass of encapsulated ciprofloxacin in μg per 1 mg of loaded nanoparticles **C-L-NPs-C-CS-I-M**, **C-L-NPs-C-CS-II-M** or **C-L-NPs-C-CS-III-M**). The characteristics of the loaded nanoparticles are presented in Table 7. The loaded nanoparticles are redispersible after lyophilization and they are stable in suspension at least 48 h.

**Figure 5:**
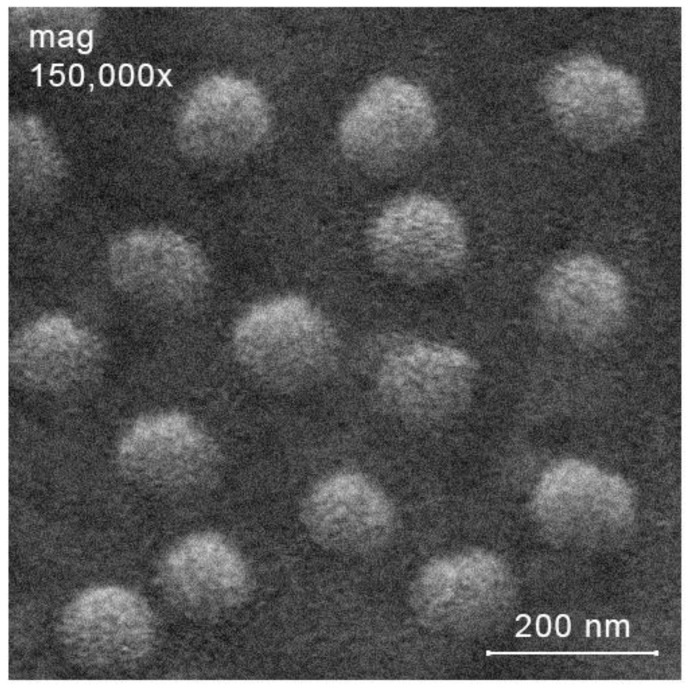
SEM image of loaded self-assembled nanoparticles.

The loaded nanoparticles do not dramatically differ in their sizes as compared with the starting (unloaded) nanoparticles. However, it can be seen that the difference in the sizes of loaded and unloaded nanoparticles increases with an increase in the degree of substitution of the polymer used. For example, the difference in hydrodynamic diameter for unloaded and loaded nanoparticles derived from **C-CS-I-M** (degree of substitution 0.05) is ca. 9 nm, while this value between loaded and unloaded nanoparticles derived from **C-CS-III-M** (degree of substitution 0.20) is ca. 27 nm. The scanning electron microscopy results (see, for example, Figure 5) indicated the formation of spherical nanoparticles and confirmed the hydrodynamic diameter values obtained by dynamic light scattering analysis. The ξ-potential of the loaded nanoparticles is positive and its value in mV is closed to that for the starting (unloaded) nanoparticles. The loaded ciprofloxacin content in the nanoparticles was in the range ca. 264–317 μg/mg and increased pronouncedly with the increase of degree of substitution of the starting polymer. 

#### 2.3.2. Preparation of Ciprofloxacin-Loaded Nanoparticles from Other Polymers by Ionic Gelation Method (Scheme 1, System 4 and Scheme 1, System 6)

**Figure 6:**
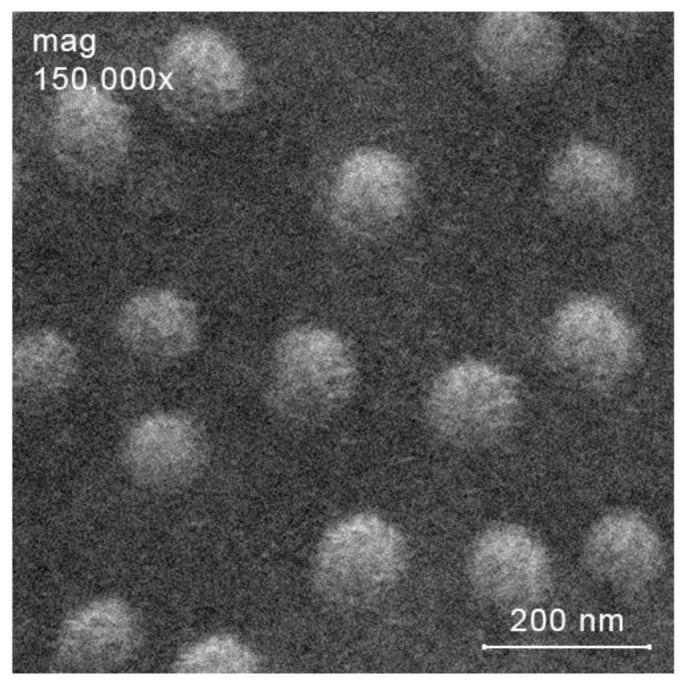
SEM image of the loaded nanoparticles prepared by ionic gelation method.

The obtained nanoparticles are characterized by positive ζ-potential value. The hydrodynamic diameter of the resulting nanoparticles naturally increases with an increase in the degree of substitution and the molecular weight of the starting polymers. It should be noted that in almost all cases, nanoparticles prepared from conjugates in which ciprofloxacin is bound to the polymer chain through the spacer are large compared to nanoparticles obtained from conjugates in which ciprofloxacin is directly bound to the polymer. In addition, from the example of *loaded* nanoparticles **C-L-NPs-C-SP-CS-I-M**, **C-L-NPs-C-SP-CS-II-M** and **C-L-NPs-C-SP-CS-III-M** (see Table 8) can be observed that they are characterized by a larger size than *the corresponding unloaded* nanoparticles **NPs-C-SP-CS-I-M**, **NPs-C-SP-CS-II-M** and **NPs-C-SP-CS-III-M** (see Table 6). Loading efficiency of the loaded nanoparticles obtained by ionic gelation method are in the range 78–98 μg/mg, and these values are significantly less than those characteristics of related self-assembled nanoparticles described in Table 7. Example of SEM image of the loaded nanoparticles prepared by ionic gelation method (**C-L-NPs-C-SP-CS-III-M**) is presented in Figure 6.

#### 2.3.3. Drug Release Study

In this section, we evaluated drug release from the following systems:

(i) ciprofloxacin-chitosan conjugates without pH-sensitive spacer (Scheme 1, system 1; Table 2);
(ii) ciprofloxacin-chitosan conjugates with the spacer (Scheme 1, system 5; Table 5);
(iii) loaded self-assembled nanoparticles (based on ciprofloxacin-chitosan conjugates without the spacer, Scheme 1, system 3; Table 7);
(iv) loaded nanoparticles based on ciprofloxacin-chitosan conjugates without the spacer prepared by ionic gelation method (Scheme 1, system 4; Table 8, part I);
(v) loaded nanoparticles based on ciprofloxacin-chitosan conjugates with the spacer prepared by ionic gelation method (Scheme 1, system 6; Table 8, part II).

**Figure 7:**
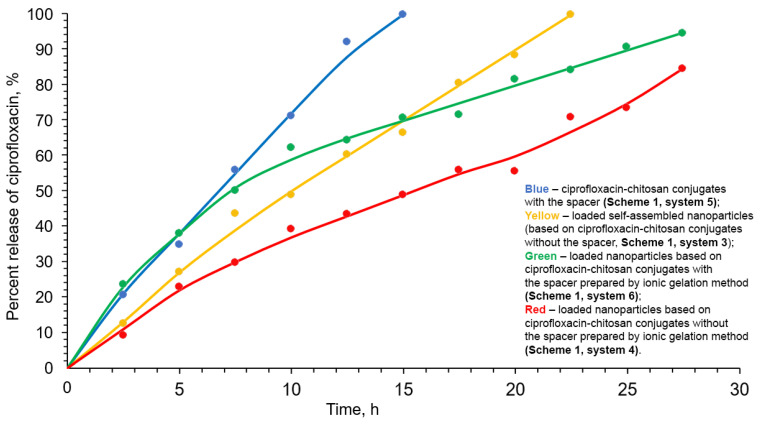
Ciprofloxacin release profiles from systems based on chitosan of moderate molecular weight (6.9 × 10^4^ (**M**), see Section 2.1).

The experiments were carried out at 40 °C and at pH value characteristic of the inflammatory process of bacterial etiology (6.5). The experimental results (the so-called release profiles) are summarized in Figure 7 as the percentage of ciprofloxacin released versus time. The triplicate experiments showed that conjugates without the spacer, independently of molecular mass and degree of substitution of the tested conjugate, do not release ciprofloxacin during at least 80 h. Ciprofloxacin-chitosan conjugates with the spacer are characterized by fast and fairly uniform release kinetics and release 100% of the drug within 15 h. Loaded self-assembled nanoparticles (based on ciprofloxacin-chitosan conjugates without the spacer) have a similar release profile, however, the release is somewhat slower and reaches 100% in 20 h. Loaded nanoparticles prepared by ionic gelation method are also characterized by similar release profiles among themselves. However, nanoparticles based on conjugates without the spacer release the drug somewhat more slowly and have a more uniform release profile. Figure 7 shows the results for systems based on medium molecular weight chitosan. For the studied samples based on chitosan of lower and higher molecular weight, no differences were found compared to the results presented in Figure 7. Figure 7 displays the results for degree of substitution 0.20. For lower degrees of substitution (0.10 and 0.05), a slightly faster release is characteristic (by 5–10%). This can be explained by the decrease in the content of hydrophobic fragment in the polymer molecule, which would bind and retain ciprofloxacin. The error function analysis is presented in Section 2.4.

### 2.4. Antibacterial Activity of Nanoparticles and Conjugates In Vitro

The in vitro activity of the prepared fluoroquinolone/chitosan-based antibacterial systems (conjugates and nanoparticles) was evaluated using agar diffusion method. The results of the study are presented in Table 9.

All tested antibacterial systems are characterized by a higher antibacterial effect than that of the starting chitosan. Among conjugates, the lowest antibacterial activity was observed for the ciprofloxacin-chitosan conjugates without the spacer. The antibacterial effect of the conjugates increases with the increase in their degree of substitution, which means that the grafted ciprofloxacin is the pharmacophore that makes an important (if not major) contribution to the antibacterial action of the conjugate. Increased antibacterial activity of ciprofloxacin-chitosan conjugates with the spacer can be explained by their ability to release free ciprofloxacin, and thereby to provide the symbaticity of the mechanisms of action characteristic of both free ciprofloxacin (inhibition of DNA gyrase and topoisomerase IV [36]) and chitosan polymer chain (damage to the bacterial cell membrane [37]).

In many instances, polymer-based nanoparticles are characterized by significantly improved antibacterial activity as compared with the polymers in their native form [38]. The mechanisms of the antibacterial effect of nanoparticles are poorly understood, and three modes of nanoparticle-mediated antibacterial action are considered. Nanoparticles with positive ζ-potential value can (i) strongly bind to anionic moieties of bacterial cell membrane, causing its irreversible damage; (ii) penetrate the bacterial cell and bind to polyanionic DNA leading to inhibition of its replication and protein synthesis; and (iii) cause cells to coagulate. This cascade of events unfavorable for the microbial cell leads to its inevitable death [39]. In this study, all ciprofloxacin-loaded nanoparticles demonstrated significantly higher antibacterial effect than the related conjugates. The most active antibacterial agents proved to be ciprofloxacin-loaded self-assembled nanoparticles based on ciprofloxacin-chitosan conjugates without the spacer. Their antibacterial effect against both *S. aureus* and *E. coli* is comparable with that of the reference antibiotic ciprofloxacin (MIC for *E. coli*: ciprofloxacin 0.10 µg/mL, **C-L-NPs-C-CS-I-M** 0.13 µg/mL, **C-L-NPs-C-CS-II-M** 0.11 µg/mL, **C-L-NPs-C-CS-III-M** 0.10 µg/mL; MIC for *S. aureus*: ciprofloxacin 2.10 µg/mL, **C-L-NPs-C-CS-I-M** 2.60 µg/mL, **C-L-NPs-C-CS-II-M** 2.40 µg/mL, **C-L-NPs-C-CS-III-M** 2.05 µg/mL).

Recent studies demonstrated that poly-cations and positively charged nanoparticles change membrane permeability and provoke membrane rapture resulting in the loss of a range of intracellular compounds (proteins, lactate dehydrogenase, ribonucleic and deoxyribonucleic acids) [40]. Spectrophotometry of a suspension of bacterial cells in a 0.5% aqueous solution of NaCl in the UV region is a very convenient way of monitoring integrity of bacterial cell membrane. This method is based on the fact that bacterial intracellular components are characterized by strong absorption at 260 nm. Using this approach, we evaluated the effect of (i) chitosan, (ii) ciprofloxacin, (iii) loaded self-assembled nanoparticles based on ciprofloxacin-chitosan conjugates without the spacer (the most effective developed in this project antibacterial system) on the integrity of the cell membranes of *S. aureus* as the model microorganism.

**Figure 8:**
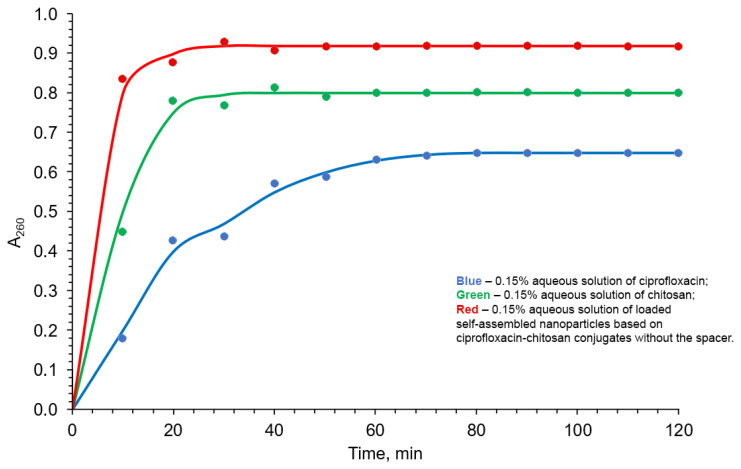
The release of the contents of bacterial cells that absorb at 260 nm from *S. aureus* under treatment of bacterial suspension with an appropriate solution.

Figure 8 demonstrates that ciprofloxacin does cause disruption of the membrane permeability of the bacterial cell of *S. aureus*, but to a lesser extent than chitosan. The greatest effect of releasing the bacterial cell contents is caused by the loaded self-assembled nanoparticles based on ciprofloxacin-chitosan conjugates without the spacer. Apparently, this is due to the summation of the action of ciprofloxacin and the positively charged nanoparticles, which are capable of damaging the membrane of a bacterial cell. Table 10 represents the error function analysis.

### 2.5. Antibacterial Activity of the Loaded Self-Assembled Nanoparticles Based on Ciprofloxacin-Chitosan Conjugates without the Spacer In Vivo

Based on results described in the previous sections, for the in vivo experiments, we chose the loaded self-assembled nanoparticles based on ciprofloxacin-chitosan conjugates without the spacer as the most promising antibacterial systems among those tested in the current study. We tested the nanoparticles in vivo on white Wistar rats in comparison with the starting chitosan and ciprofloxacin. For the experiments, we subjected the rats to the conventional model peritonitis, using a microbial mixture containing *S. aureus* and *E. coli* as infecting agents.

Six hours after the administration of the microorganisms, all rats demonstrated the classical symptoms of peritonitis: lethargy, refusal to eat, rapid breathing, bloating. In the control groups, exudate collection (200 μL) was performed with a sterile syringe 24 h after the infection. After a day, all other rats were injected with a nanosuspension of the tested nanoparticles, or chitosan, or ciprofloxacin. After 7 h 200 μL of exudate were taken. To 200 μL of the exudate, 1000 μL of 0.9% aqueous NaCl was assed. 100 μL of the formed solution was applied uniformly to a Petri dish with meat-peptone agar. Colony counting was performed 24 h after incubation in an incubator at 37 °C. Subsequently, the colony-forming unit (CFU) was recalculated per 1 mL of exudate.

The results of the in vivo experiments are presented in Table 11. The lowest in vivo antibacterial activity was observed for the starting chitosan. The use of chitosan as antibacterial agent led to the value of CFU per 1 mL of exudate was only ca. 1.5 times less than that of the blank experiment. As the blank experiment peritonitis-infected probe without treatment was used. A fairly high activity displayed ciprofloxacin. The tested nanoparticles demonstrated an extremely high in vivo antibacterial effect, and we detected no growth of colonies was after the exudate sampling. The high activity of the nanoparticles is due to good their release profile. Moreover, the lower efficacy of the ciprofloxacin could be explained by its fast elimination. The elimination of the nanoparticles proceeds much more difficult, and this could result in an increase in the antibacterial effect of the nanoparticles compared to ciprofloxacin.

### 2.6. Toxicity of the Loaded Self-Assembled Nanoparticles Based on Ciprofloxacin-Chitosan Conjugates without the Spacer

Recent studies demonstrated that in many instances chitosan-based antibacterial systems are characterized low toxicity and biodegradability [41]. In particular, conjugation of a toxic antibacterial pharmacophore to chitosan backbone could overcome its toxicity without the loss of the antibacterial activity [42]. In this study, we evaluated the in vitro toxicity of the most promising nanoparticles (**C-L-NPs-C-CS-I-M**, **C-L-NPs-C-CS-II-M** and **C-L-NPs-C-CS-III-M**) using the conventional MTT-test. MTT test is a colorimetric method for evaluation the percentage of viable cells in culture. The basis of this method is that NADPH-dependent dehydrogenases of viable cells efficiently reduce 3-(4,5-dimethylthiazol-2-yl)-2,5-diphenyl-tetrazolium bromide (MTT) to give formazan of purple color. Thus, the intensity of the purple color corresponds to the cell viability [28]. The results demonstrated that at a concentration of 10 μg/mL (much more than MIC of both ciprofloxacin and the tested nanoparticles), the cell viability with action of the nanoparticles was ca. 95%, while under ciprofloxacin the cell viability was ca 80%.

We also evaluated the in vivo toxicity of the prepared nanoparticles in rats. We found that a single injection of nanosuspension of **C-L-NPs-C-CS-I-M**, **C-L-NPs-C-CS-II-M** or **C-L-NPs-C-CS-III-M** at a dose of 2000 mg/kg does not affect the general condition of the animals, symptoms of acute poisoning were not recorded, and there was no death of rats. Since the studied dose of the drug did not lead to the death of a single animal, it was concluded that LD_50_ > 2000 mg/kg and the tested nanoparticles belong to the IV class of hazard (low-toxic substances). During the entire observation period, the behavioral reactions of the animals in the experimental group were within the physiological norm: normal drinking and eating behavior, normal coordination of movements, the usual frequency and depth of respiratory movements, normal consistency of fecal masses, frequency of urination and color of urine were noted. During this time, the rats gained weight. Hematological parameters of peripheral blood during the experiment changed slightly within the normal range (Table 12, Table 13 and Table 14).

Thus, based on the experimental data of both in vitro and in vivo studies, we can conclude, that the leading antibacterial chitosan-based systems **C-L-NPs-C-CS-I-M**, **C-L-NPs-C-CS-II-M** and **C-L-NPs-C-CS-III-M** are non-toxic.

## 3. Materials and Methods

### 3.1. Raw Materials

In this study, we used crab shell chitosan (Bioprogress, Shchyolkovo, Russia) with a viscosity-average molecular weight (MW) of 3.7 × 10^4^ (**L**) and a degree of acetylation of 26%, viscosity-average molecular weight (MW) of 6.9 × 10^4^ (**M**) and a degree of acetylation of 28% and viscosity-average molecular weight (MW) of 17.8 × 10^4^ (**H**) and a degree of acetylation of 24%, ciprofloxacin, *n-*butylamine, DCC (*N,N′-*dicyclohexylcarbodiimide), NHS (*N-*hydroxysuccinimide), hydrazine, glyoxal, TPP (sodium tripolyphosphate) (Sigma Aldrich, Burlington, MA, USA). Other chemicals and solvents were obtained from commercial sources and used as received without further purification.

### 3.2. General Methods

Thin layer chromatography (TLC) was performed on Merck 60 F_254_SiO_2_ plates with hexane:chloroform 1:1 (*v*:*v*) mixture as an eluent.

The ^1^H NMR spectra were recorded on a Bruker spectrometer (Ettlingen, Germany) operating at a frequency of 400 MHz. 

High-resolution electrospray ionization mass spectrometry (positive ion mode) was carried out on a Bruker APEX-Qe ESI FT-ICR instrument (Daltonics, DE, USA) with CH_3_CN as a solvent.

The apparent hydrodynamic diameter and ζ-potential of nanoparticles in water were estimated at room temperature (about 20 °C) using a Photocor Compact-Z instrument (Russia) at λ = 659 nm and θ = 90°.

**Figure 9:**
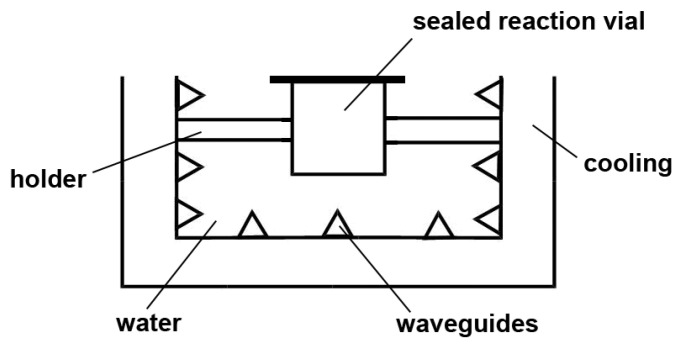
Principal scheme of experimental setup.

Ultrasonic treatments were carried out in an ultrasonic bath (USB300X, ITA) equipped by temperature control device. The ultrasonic bath can work at frequencies of 22 kHz, 30 kHz, 45 kHz, 70 kHz, 80 kHz, 100 kHz, 250 kHz, 270 kHz, 280 kHz, 290 kHz, or 300 kHz with a variable power output from 120 W to 300 W. The ultrasonic energy was delivered from the bottom of the bath to water by six coupled transducers. For each experimental run, the reaction mixture was loaded into a tube, and then placed in the water bath and fixed at the same position during the ultrasound treatment (Figure 9).

Elemental analyses were carried out using a Perkin-Elmer elemental analyzer.

SEM images were obtained by electron microscope JEOL JSM—6490 LV at 15 kV, SEM detector, electron beam size 30, in high vacuum. The test samples were coated with 20 nm (40 s at 40 mA) with a platinum layer in a JEOL auto fine coater JFC—1600.

UV spectra were recorded using Mettler UV5 spectrophotometer.

### 3.3. Synthetic Work

#### 3.3.1. Model Reaction of Ciprofloxacin with n-Butylamine

0.1 g of ciprofloxacin was dissolved in 1% aqueous solution of acetic acid (10 mL) and then 1.1 equiv. of n-butylamine, 1.3 equiv. of DCC and 1.3 equiv. of NHS were added. The reaction mixture was sonicated at 22–300 kHz and 120–300 W. The reaction was monitored by TLC. The product was purified by column chromatography (SiO_2_, 40–60 Mash, chloroform/acetone 15/1, *v*/*v*).

Found for C_21_H_24_FN_4_O_2_, %: C 65.35, H 7.02, N 14.56 (calculated, %: C 65.27, H 7.04, N 14.50); HRESI-MS, *m/z*: 387.2194, [M + H]^+^ (calculated 387.2196); ^1^H NMR, δ, CD_3_OD: 0.91, m, 3H, 1.06–1.48 3 m, 6H, 2.77, t, 4H, 3.23, t, 2H, 3.49, t, 4H, 4.15, m, 1H, 6.05, d, 1H, 8.00, d, 1H, 9.01, s, 1H.

#### 3.3.2. Synthesis of Ciprofloxacin-Chitosan Conjugates in Which Ciprofloxacin Is Directly Attached to the Polymer Chain (without the Spacer, Scheme 1, System 1; Table 2)

Under ultrasound-free conditions, 0.5 g of chitosan was dissolved in 1% acetic acid (20 mL), the pH of the solution was adjusted to 3; then 0.5, 1.8 or 6.5 equiv. of ciprofloxacin, DCC and NHS were added, and the reaction mixtures were stirred at 50 °C for 5 h. The formed polymers were precipitated by addition of 25 mL of acetone. The precipitated polymers were dissolved in water, dialyzed against distilled water and freeze-dried.

The synthesis of the mentioned above chitosan derivatives under ultrasonic conditions differs from that under ultrasound-free conditions in that 0.3, 1.0 or 3.5 equiv. of ciprofloxacin, EDC and NHS were added, and the reaction mixtures were treated with ultrasonic irradiation at 100 kHz, 280 W for 20 min at 50 °C.

To determine the degree of substitution, the signal of anomeric protons of all types of units (***1***,***1***′,***1***″) was chosen as the reference signal (I(***1***,***1***′,***1***″) = 1). The degree of substitution was calculated as DS = I(***1***′). I—integral intensities of the corresponding signals. Signal assessments with proton numbering are given in Figure 1.

#### 3.3.3. Synthesis of Ciprofloxacin-Chitosan Conjugates in Which Ciprofloxacin Is Linked to the Polymer Chain through the pH-Sensitive Linker (Scheme 1, System 5; Table 5)

##### Synthesis of Hydrazone 1 (Scheme 4, A)

Ciprofloxacin (0.5 g) was dissolved in methanol (10 mL), then 2 equiv. of hydrazine hydrate and 1 drop of triflouroacetic acid were added. The reaction mixture was stirred for 6 h. The solvent was removed under vacuo, and the resulting product was purified by column chromatography (SiO_2_, 40–60 Mash, hexane/ethyl acetate 8/1, *v*/*v*).

Found for C_17_H_20_FN_5_O_2_, %: C 59.18, H 5.87, N 20.23 (calculated, %: C 59.12, H 5.84, N 20.25); HRESI-MS, *m/z*: 346.1675, [M + H]^+^ (calculated 346.1679); ^1^H NMR, δ, CD_3_OD: 1.09 and 1.37 two m, 4H, 2.80, t. 4H, 3.45, t, 4H, 4.14, m, 1H, 5.89, s, 1H, 6.00, br s, 2H, 7.50, s, 1H, 8.40, s, 1H, 15.10 br s, 1H.

##### Synthesis 2 (Scheme 4, B)

Hydrazone **1** was dissolved in methanol, and 1 drop of triflouroacetic acid and 3.5 equiv. of glyoxal were added. The reaction mixture was stirred 5 h. The solvent was removed under vacuo, and the resulting product was purified by column chromatography (SiO_2_, 40–60 Mash, hexane/ethyl acetate 10/1, *v*/*v*).

Found for C_19_H_20_FN_5_O_3_, %: C 59.23, H 5.26, N 18.15 (calculated, %: C 59.21, H 5.23, N 18.17); HRESI-MS, *m/z*: 386.1624, [M + H]^+^ (calculated 386.1628); ^1^H NMR, δ, CD_3_OD: 1.09 and 1.37 two m, 4H, 2.80, t. 4H, 3.45, t, 4H, 4.15, m, 1H, 5.90, s, 1H, 7.52, s, 1H, 8.25, s, 1H, 8.45, s, 1H, 9.70, s, 1H, 15.11 br s, 1H.

##### Conjugation of 2 with Chitosan (Scheme 4, C)

Under ultrasound-free conditions, 0.5 g of chitosan was dissolved in 1% acetic acid (20 mL), the pH of the solution was adjusted to 3; then 0.9, 1.7 or 3.5 equiv. **2** were added, and the reaction mixtures were stirred at 15 °C for 3 h. The formed polymers were precipitated by addition of 25 mL of acetone. The precipitated polymers were washed by acetone, methanol ethanol and dried in vacuo.

The synthesis of the mentioned above chitosan derivatives under ultrasonic conditions differs from that under ultrasound-free conditions in that 0.5, 0.9 or 1.6 equiv. of **2** added, and the reaction mixtures were treated with ultrasonic irradiation at 80 kHz, 250 W for 20 min at 25 °C.

To determine the degree of substitution, the signal of anomeric protons of all types of units (***1***,***1***′,***1***″) was chosen as the reference signal (I(***1***,***1***′,***1***″) = 1). The degree of substitution was calculated as DS = I(***1***′). I—integral intensities of the corresponding signals. Signal assessments with proton numbering are given in Figure 3.

#### 3.3.4. Preparation of Unloaded Nanoparticles

Self-assembled nanoparticles (Scheme 1, system 2; Table 3) were prepared as follows. **C-CS-I-M**, **C-CS-II-M** or **C-CS-III-M** were dissolved in 1% acetic acid in concentration 1 mg/mL, pH of the solution was adjusted to 7.0 by addition of sodium hydrocarbonate. The suspension was sonicated for 5 min (30 kHz, 300 W). The resulting nanoparticles were centrifuged, washed by water followed by centrifugation (3 times), redispersed in water and freeze dried.

Nanoparticles described in Table 6 were prepared by dissolving 20 mg of **C-SP-CS-I-M**, **C-SP-CS-II-M** or **C-SP-CS-III-M** in 20 mL of 1% acetic acid. After 3 h of stirring, 3.2, 2.1 or 1.5 mL of 0.25% aqueous sodium tripolyphosphate (TPP) solution was rapidly added. The resulting nanoparticle suspension was centrifuged, washed by water followed by centrifugation (3 times), redispersed in water and freeze dried.

#### 3.3.5. Preparation of Loaded Nanoparticles

Loaded self-assembled nanoparticles (Scheme 1, system 3; Table 7) were prepared as follows. A total of 5 mg of ciprofloxacin dissolved in ethanol (1 mL) was added to 10 mg of **NPs-C-CS-I-M**, **NPs-C-CS-II-M** or **NPs-C-CS-III-M** suspended in water (5 mL) followed by sonication at 22 kHz, 120 W for 35 s. Then the nanoparticles were centrifuged to separate them from the supernatant. The separated nanoparticles were washed by water and freeze dried. Loading efficiency (LE) and encapsulation efficiency (EE) of the loaded nanoparticles were calculated using the following equations:

LE = [(m_(ciprofloxacin total)_ − m_(ciprofloxacin in supernatant)_)/m_(ciprofloxacin total)_] × 100

EE = [(m_(ciprofloxacin total)_ − m_(ciprofloxacin in supernatant)_)/m_(loaded nanoparticles)_] × 100

The mass of ciprofloxacin in supernatant was determined by UV-spectroscopy at a wavelength of 278 nm after addition of HCl to final concentration 0.1 M (calibration curve method). Nanoparticles (Scheme 1, system 4; Table 8 part 1) and nanoparticles (Scheme 1, system 6; Table 8 part 2) were prepared by dissolving 10 mg of the corresponding polymer in 5 mL of 1% acetic acid. Then 5 mg of ciprofloxacin in ethanol was added and 0.25% sodium TPP solution (volume see in Table 8) was rapidly added under vigorous stirring. Then the nanoparticles were centrifuged to separate them from the supernatant. The separated nanoparticles were washed by water and freeze dried. To evaluate the loading efficiency (LE) and the encapsulation efficiency (EE), after centrifugation of nanoparticles dispersion at 7500 rpm for 40 min, 5 mL of 10 mg/mL calcium chloride solution was added separately to both the pellet and the supernatant. The resulting mixture was stirred overnight, the resulting calcium tripolyphosphate precipitated, and the mixture was centrifuged at 7500 rpm for 15 min and analyzed by UV spectroscopy using calibration curve method at 278 nm. LE and EE values were calculated as described above.

### 3.4. Drug Release Kinetics Study

A 1 mg sample of nanoparticles or conjugate was dispersed or dissolved in buffer solution (5 mL, pH 6.5) and incubated at 32 °C with gentle stirring. At regular intervals, to 2 mL of medium was added 0.5 mL of water (in the case of nanoparticles) or 0.5 mL of 0.25% sodium TPP (in the case of conjugates) were added. The resulting mixtures were ultracentrifuged at 4500 rpm. The amount of released ciprofloxacin was in the supernatant determined by UV spectroscopy using calibration curve method at 278 nm.

### 3.5. Antibacterial Activity

The in vitro antibacterial activity of the chitosan-based nanoparticles was evaluated by the agar well diffusion method [42,43,44]. The antibacterial effect was studied against *Staphylococcus aureus* (RCMB 010027) and *Escherichia coli* (RCMB 010051). The activity was determined by measuring the diameter of the inhibition zone (in mm). Each inhibition zone was measured three times by a caliper to get an average value. Ampicillin and gentamicin were used as reference antibacterial drugs [45].

The in vivo antibacterial activity (in white rats) was performed as described elsewhere [46]. Animal procedures were compliant with the Ethics Committee and followed the recommendations of European Directive 2010/63/EU of 22 September 2010.

### 3.6. Toxicity Study

The in vitro toxicity was estimated by the MTT test. Solutions or nanosuspensions of the tested samples were prepared by serial dilution in alpha-MEM culture medium. A 0.1 mL volume of each of nanosuspension was added to a confluent monolayer of cells cultured in a 96-well plate. Cells were incubated for 24 h at 37 °C in an atmosphere containing 5% CO_2_. The cells were washed twice with PBS and then 0.1 mL of 3-(4,5-dimethylthiazolyl)-2,5-diphenyltetrazolium bromide (MTT, 0.5 μg/mL) in PBS was added and the mixture was incubated for 4 h. The supernatant was then replaced with 0.1 mL of 96% ethanol, and the absorbance was measured at 535 nm.

The in vivo toxicity in white rats was studied as described elsewhere [46]. Animal procedures were compliant with the Ethics Committee and followed the recommendations of European Directive 2010/63/EU of 22 September 2010.

### 3.7. Statistical Analysis

The statistical significance of differences between the samples was determined by a one-way analysis of variance (ANOVA) using JMP 5.0.1 software (SAS Campus Drive, Cary, NC, USA). Mean values, where appropriate, were compared by applying the Student’s *t-*test at a significance level *p* < 0.05.

## 4. Conclusions

The results of this work can be considered from the following main perspectives.

1. We demonstrated that DCC-mediated coupling between COOH and NH_2_ groups can be efficiently promoted by ultrasound in water. Using this approach, we successfully prepared the first ciprofloxacin-chitosan conjugates, which do not contain any spacer.
2. We synthesized the first ciprofloxacin-chitosan conjugates carrying an antibiotic, which is attached to the polymer chain through a pH-sensitive spacer.
3. Thirdly, we elaborated three types of loaded by the ciprofloxacin nanoparticles based on both conjugates with or without a spacer (Scheme 1).
  Ciprofloxacin-loaded self-assembled nanoparticles, based on conjugates without any spacer, demonstrated to be capable to release the antibiotic featuring the best release profile. Moreover, their in vitro antibacterial effect is the best among all chitosan/ciprofloxacin-based systems regarded in the current work.
4. We evaluated the in vivo antibacterial activity and the in vivo acute and sub-acute toxicity of the best-performing antibacterial nanoparticles (self-assembled ciprofloxacin-loaded nanoparticles based on conjugates without any spacer). The in vivo antibacterial effect of the nanoparticles exceeded even that of the starting ciprofloxacin. Moreover, the in vivo toxicity of the nanoparticles was almost identical to that of the chitosan, which is considered as the non-toxic biopolymer.
5. The obtained results inspired us to regard the elaborated leading nanoparticles as the systems which are of interest for prolonged release of ciprofloxacin and its targeted delivery. The developed nanoparticles need to be turned into a suitable dosage form, as well as in further in vivo pharmacological experiments and this project is underway in our group.

## Figures and Tables

**Table 1:** Effect of ultrasonic irradiation on the synthesis of ciprofloxacin/chitosan conjugate without spacer (Scheme 1, system 1).

Parameters, Identical for Both Ultrasound-Free and Ultrasonic Conditions			Ultrasound-Free Conditions		Ultrasonic Conditions	
DS	T, °C	pH	Molar Ratio	Reaction Time	Molar Ratio	Reaction Time
0.05	50 °C	3	0.5	5 h	0.2	20 min
0.10	50 °C	3	1.5	5 h	0.5	20 min
0.20	50 °C	3	4	5 h	1.6	20 min

**Table 2:** Degrees of substitution of the obtained of ciprofloxacin-conjugated chitosan derivatives without spacer (Scheme 1, system 1) and molecular weights of the starting chitosans.

Ciprofloxacin/Chitosan Conjugate	The Molecular Weight of the Starting Chitosan	The Degree of Substitution of the Derivative
**C-CS-I-L**	3.5 × 10^4^	0.05
**C-CS-I-M**	7.1 × 10^4^	0.05
**C-CS-I-H**	17.2 × 10^4^	0.05
**C-CS-II-L**	3.5 × 10^4^	0.10
**C-CS-II-M**	7.1 × 10^4^	0.10
**C-CS-II-H**	17.2 × 10^4^	0.09
**C-CS-III-L**	3.5 × 10^4^	0.20
**C-CS-III-M**	7.1 × 10^4^	0.20
**C-CS-III-H**	17.2 × 10^4^	0.20

**Table 3:** Characteristics of the self-assembled nanoparticles (Scheme 1, system 2).

Sample	2R_h_, nm *	PDI *	ζ-Potential, mV *
**NPs-C-CS-I-M**	35 ± 5	0.18 ± 0.03	32.4 ± 0.3
**NPs-C-CS-II-M**	59 ± 3	0.16 ± 0.02	35.2 ± 0.1
**NPs-C-CS-III-M**	97 ± 3	0.18 ± 0.02	39.7 ± 0.3

* Mean value ± SD, *n* = 3.

**Table 4:** Effect of ultrasonic irradiation on synthesis of ciprofloxacin conjugate with chitosan, containing the pH sensitive spacer.

Parameters, Identical for Both Ultrasound-Free and Ultrasonic Conditions			Ultrasound-Free Conditions		Ultrasonic Conditions	
DS	T, °C	pH	Molar Ratio	Reaction Time	Molar Ratio	Reaction Time
0.05	25 °C	3	0.9	3 h	0.5	20 min
0.10	25 °C	3	1.7	3 h	0.9	20 min
0.20	25 °C	3	3.5	3 h	1.6	20 min

**Table 5:** Degrees of substitution of the obtained of ciprofloxacin-conjugated chitosan derivatives with the pH sensitive spacer (Scheme 1, system 5) and molecular weights of the starting chitosans.

Ciprofloxacin/Chitosan Conjugate	The Molecular Weight of the Starting Chitosan	The Degree of Substitution of the Derivative
**C-SP-CS-I-L**	3.5 × 10^4^	0.06
**C-SP-CS-I-M**	7.1 × 10^4^	0.05
**C-SP-CS-I-H**	17.2 × 10^4^	0.04
**C-SP-CS-II-L**	3.5 × 10^4^	0.11
**C-SP-CS-II-M**	7.1 × 10^4^	0.10
**C-SP-CS-II-H**	17.2 × 10^4^	0.09
**C-SP-CS-III-L**	3.5 × 10^4^	0.22
**C-SP-CS-III-M**	7.1 × 10^4^	0.20
**C-SP-CS-III-H**	17.2 × 10^4^	0.20

**Table 6:** Characteristics of the nanoparticles prepared by ionic gelation method.

Sample	2R_h_, nm *	PDI *	ζ-Potential, mV *
**NPs-C-SP-CS-I-M**	39 ± 5	0.11 ± 0.02	31.0 ± 0.4
**NPs-C-SP-CS-II-M**	67 ± 3	0.14 ± 0.03	37.9 ± 0.3
**NPs-C-SP-CS-III-M**	110 ± 4	0.15 ± 0.03	40.3 ± 0.2

* Mean value ± SD, *n* = 3.

**Table 7:** Characteristics of loaded self-assembled nanoparticles (Scheme 1, system 3).

Sample	2R_h_, nm *	PDI *	ζ-Potential, mV *	EE, % *	LE, μg/mg * (%)
**C-L-NPs-C-CS-I-M**	48 ± 3	0.13 ± 0.04	33.0 ± 0.5	73 ± 5	264 ± 3 (ca. 26%)
**C-L-NPs-C-CS-II-M**	83 ± 4	0.15 ± 0.03	38.4 ± 0.2	81 ± 2	283 ± 5 (ca. 28%)
**C-L-NPs-C-CS-III-M**	137 ± 2	0.12 ± 0.02	40.0 ± 0.4	93 ± 3	317 ± 2 (ca. 32%)

* Mean value ± SD, *n* = 3.

**Table 8:** Characteristics of loaded nanoparticles obtained by ionic gelation method (Scheme 1, system 4 and Scheme 1, system 6).

Sample	Volume of TPP (mL)	2R_h_, nm *	PDI *	ζ-Potential, mV *	EE, % *	LE, μg/mg * (%)
***Part I*** *(nanoparticles based on the conjugates without the spacer, (* Scheme 1 *, system 4))*						
**C-L-NPs-C-CS-I-L**	3.0	43 ± 2	0.09 ± 0.02	34.3 ± 0.1	31 ± 3	80 ± 2
						(ca. 8%)
**C-L-NPs-C-CS-I-H**	3.0	49 ± 3	0.12 ± 0.03	33.2 ± 0.5	32 ± 4	80 ± 2
						(ca. 8%)
**C-L-NPs-C-CS-II-L**	2.0	63 ± 3	0.13 ± 0.04	38.2 ± 0.3	28 ± 2	82 ± 3
						(ca. 8%)
**C-L-NPs-C-CS-II-H**	2.0	69 ± 1	0.10 ± 0.03	39.1 ± 0.1	30 ± 3	91 ± 2
						(ca. 9%)
**C-L-NPs-C-CS-III-L**	1.5	128 ± 4	0.15 ± 0.02	43.3 ± 0.2	27 ± 6	88 ± 4
						(ca. 9%)
**C-L-NPs-C-CS-III-H**	1.5	126 ± 3	0.12 ± 0.03	45.8 ± 0.3	30 ± 3	98 ± 2
						(ca. 10%)
***Part II****(nanoparticles based on the conjugates with the spacer,* Scheme 1*, system 6)*						
**C-L-NPs-C-SP-CS-I-L**	3.0	48 ± 2	0.12 ± 0.04	32.9 ± 0.3	31 ± 5	78 ± 4
						(ca. 8%)
**C-L-NPs-C-SP-CS-I-M**	3.0	51 ± 3	0.14 ± 0.03	33.1 ± 0.4	33 ± 5	81 ± 2
						(ca. 8%)
**C-L-NPs-C-SP-CS-I-H**	3.0	59 ± 2	0.12 ± 0.03	32.5 ± 0.2	30 ± 3	78 ± 8
						(ca. 8%)
**C-L-NPs-C-SP-CS-II-L**	2.0	64 ± 4	0.16 ± 0.02	35.3 ± 0.4	27 ± 4	80 ± 4
						(ca. 8%)
**C-L-NPs-C-SP-CS-II-M**	2.0	66 ± 2	0.12 ± 0.02	36.4 ± 0.1	31 ± 1	87 ± 1
						(ca. 9%)
**C-L-NPs-C-SP-CS-II-H**	2.0	71 ± 3	0.14 ± 0.05	36.9 ± 0.2	30 ± 2	84 ± 2
						(ca. 8%)
**C-L-NPs-C-SP-CS-III-L**	1.5	118 ± 4	0.16 ± 0.02	45.8 ± 0.2	30 ± 3	97 ± 3
						(ca. 10%)
**C-L-NPs-C-SP-CS-III-M**	1.5	125 ± 3	0.13 ± 0.04	45.3 ± 0.4	28 ± 2	91 ± 3
						(ca. 9%)
**C-L-NPs-C-SP-CS-III-H**	1.5	129 ± 3	0.12 ± 0.02	46.1 ± 0.3	26 ± 4	84 ± 3
						(ca. 8%)

* Mean value ± SD, *n* = 3.

**Table 9:** Antibacterial activity of ciprofloxacin/chitosan-based conjugates and nanoparticles.

Tested Sample	Inhibition Zone, mm *	
	*E. coli*	*S. aureus*
Ciprofloxacin-chitosan conjugates without the spacer (Scheme 1, system 1; Table 2)		
**C-CS-I-M**	14.2 ± 0.3	11.3 ± 0.1
**C-CS-II-M**	16.2 ± 0.4	13.1 ± 0.2
**C-CS-III-M**	20.0 ± 0.2	14.9 ± 0.1
Ciprofloxacin-chitosan conjugates with the spacer (Scheme 1, system 5; Table 4)		
**C-SP-CS-I-M**	23.7 ± 0.4	18.4 ± 0.5
**C-SP-CS-II-M**	27.4 ± 0.1	21.6 ± 0.3
**C-SP-CS-III-M**	31.1 ± 0.1	23.9 ± 0.4
Loaded self-assembled nanoparticles (based on ciprofloxacin-chitosan conjugates without the spacer, Scheme 1, system 3; Table 6)		
**C-L-NPs-C-CS-I-M**	47.2 ± 0.1	42.5 ± 0.5
**C-L-NPs-C-CS-II-M**	47.8 ± 0.3	42.3 ± 0.4
**C-L-NPs-C-CS-III-M**	47.9 ± 0.2	42.6 ± 0.3
Loaded nanoparticles based on ciprofloxacin-chitosan conjugates without the spacer prepared by ionic gelation method (Scheme 1, system 4; Table 7, part I)		
**C-L-NPs-C-CS-I-M**	36.0 ± 0.5	30.9 ± 0.7
**C-L-NPs-C-CS-II-M**	36.5 ± 0.1	30.2 ± 0.3
**C-L-NPs-C-CS-III-M**	36.2 ± 0.4	31.1 ± 0.2
Loaded nanoparticles based on ciprofloxacin-chitosan conjugates with the spacer prepared by ionic gelation method (Scheme 1, system 6; Table 7, part II)		
**C-L-NPs-C-SP-CS-I-M**	36.3 ± 0.1	29.9 ± 0.1
**C-L-NPs-C-SP-CS-II-M**	36.9 ± 0.4	31.2 ± 0.3
**C-L-NPs-C-SP-CS-III-M**	36.4 ± 0.3	30.5 ± 0.1
Reference antibacterials		
Ciprofloxacin	43.6 ± 0.3	38.4 ± 0.5
Chitosan	8.7 ± 0.2	7.9 ± 0.4

* Mean value ± SD, *n* = 3.

**Table 10:** Error functions with their equations.

Error Functions	Equations	Drug Release Study	Antibacterial Activity of Nanoparticles and Conjugates In Vitro
Root mean square error	$RMSE=\sqrt{\frac{1}{N}\overset{N}{\underset{i=1}{\sum }}{\left(y-y^{\prime }\right)}^{2}}$	3.683	0.0130
Mean absolute percentage error	$MAPE=\frac{1}{N}\overset{N}{\underset{i=1}{\sum }}\left|\frac{y-y^{\prime }}{y}\right|*100$	6.499	2.284
Mean absolute error	$MAE=\frac{1}{N}\overset{N}{\underset{i=1}{\sum }}\left|y-y^{\prime }\right|$	2.835	0.0112
Mean Square Error	$MSE=\frac{1}{N}\overset{N}{\underset{i=1}{\sum }}{\left(\left|y-y^{\prime }\right|\right)}^{2}$	13.561	0.000169
Determination coefficient	$R^{2}=1-\frac{\frac{1}{N}\sum_{i=1}^{N}\left|y^{\prime }-y\right|}{\frac{1}{N}\sum_{i=1}^{N}\left|y^{\prime }-y_{m}\right|}$	0.976	0.994

**Table 11:** In vivo antibacterial activity of the loaded self-assembled nanoparticles based on ciprofloxacin-chitosan conjugates without the spacer (Scheme 1, system 3; Table 7) in comparison with the starting chitosan and ciprofloxacin.

Sample	CFU/mL of Exudate (7 h after Injection or 31 h after Infection)
Control without treatment (24 h after infection)	2825
Chitosan	1930
**C-L-NPs-C-CS-I-M**	0 (no growth)
**C-L-NPs-C-CS-II-M**	0 (no growth)
**C-L-NPs-C-CS-III-M**	0 (no growth)
Ciprofloxacin	175

**Table 12:** Hematological parameters of rats following oral administration of **C-L-NPs-C-CS-III-M**.

Hematological Parameters	Acute Toxicity		Subacute Toxicity	
	Control	C-L-NPs-C-CS-III-M	Control	C-L-NPs-C-CS-III-M
Male rats				
MCV (fL)	67.31 ± 3.45	66.99 ± 3.43	64.98 ± 1.41	64.87 ± 1.40
MCH (pg)	18.87 ± 0.71	18.78 ± 0.70	19.01 ± 0.57	18.79 ± 0.54
MCHC (g/dL)	28.23 ± 0.78	28.12 ± 0.74	29.22 ± 0.27	29.15 ± 0.26
RBC (×10^6^/μL)	9.09 ± 0.34	9.07 ± 0.31	8.35 ± 0.67	8.32 ± 0.65
WBC (×10^3^/μL)	8.51 ± 1.67	8.42 ± 1.59	8.03 ± 2.01	8.01 ± 1.97
Platelets (×10^3^/μL)	843.00 ± 135.94	837.00 ± 132.62	1207.00 ± 193.54	1189.00 ± 187.73
Hct (%)	63.01 ± 3.96	62.96 ± 3.87	53.74 ± 3.53	53.04 ± 3.48
Hgb (g/dL)	18.78 ± 1.45	18.64 ± 1.44	15.68 ± 1.07	15.24 ± 1.04
Female rats				
MCV (fL)	64.36 ± 3.26	64.07 ± 3.25	61.04 ± 1.26	60.94 ± 1.24
MCH (pg)	18.13 ± 0.69	17.95 ± 0.67	18.59 ± 0.49	18.45 ± 0.47
MCHC (g/dL)	27.94 ± 0.73	27.78 ± 0.71	29.45 ± 0.34	29.37 ± 0.33
RBC (×10^6^/μL)	9.01 ± 0.33	8.97 ± 0.30	9.11 ± 0.56	9.07 ± 0.55
WBC (×10^3^/μL)	5.79 ± 0.98	5.78 ± 0.96	4.62 ± 0.27	4.59 ± 0.25
Platelets (×10^3^/μL)	987.00 ± 141.76	981.00 ± 140.25	1099.00 ± 206.23	1086.00 ± 204.65
Hct (%)	58.08 ± 3.71	57.87 ± 3.67	55.97 ± 3.73	55.81 ± 3.71
Hgb (g/dL)	16.32 ± 1.32	16.27 ± 1.31	16.72 ± 1.31	16.68 ± 1.30

Mean value ± SD.

**Table 13:** Blood biochemical parameters of rats following oral administration of **C-L-NPs-C-CS-III-M**.

Blood Biochemical Parameters	Acute Toxicity		Subacute Toxicity	
	Control	C-L-NPs-C-CS-III-M	Control	C-L-NPs-C-CS-III-M
Male rats				
Albumin (g/dL)	4.20 ± 0.16	4.17 ± 0.15	3.59 ± 0.23	3.63 ± 0.24
AST (U/L)	84.00 ± 6.74	82.75 ± 6.71	97.12 ± 11.84	93.34 ± 11.56
ALT (U/L)	11.70 ± 5.78	11.50 ± 5.76	27.09 ± 6.27	26.86 ± 6.19
BUN (mg/dL)	15.41 ± 1.38	13.88 ± 1.37	13.45 ± 1.48	13.49 ± 1.50
Creatinine (mg/dL)	0.35 ± 0.05	0.29 ± 0.05	0.34 ± 0.05	0.33 ± 0.05
Glucose (mg/dL)	209.75 ± 31.46	191.50 ± 30.96	79.12 ± 10.87	93.48 ± 12.48
Total bilirubin (mg/dL)	0.30 ± 0.05	0.29 ± 0.05	0.35 ± 0.05	0.38 ± 0.05
Total protein (g/dL)	6.32 ± 0.31	6.32 ± 0.31	6.16 ± 0.27	6.21 ± 0.28
Female rats				
Albumin (g/dL)	4.60 ± 0.19	4.67 ± 0.20	4.41 ± 0.31	4.34 ± 0.30
AST (U/L)	66.75 ± 6.24	67.46 ± 6.27	79.25 ± 6.87	79.00 ± 6.82
ALT (U/L)	12.47 ± 2.48	12.54 ± 2.51	21.97 ± 6.19	21.71 ± 6.11
BUN (mg/dL)	14.01 ± 1.51	14.13 ± 1.52	16.72 ± 1.15	15.98 ± 1.03
Creatinine (mg/dL)	0.29 ± 0.05	0.29 ± 0.05	0.34 ± 0.05	0.33 ±= 0.05
Glucose (mg/dL)	159.00 ± 35.27	167.00 ± 36.74	44.50 ± 8.96	49.00 ± 9.17
Total bilirubin (mg/dL)	0.44 ± 0.05	0.45 ± 0.05	0.53 ± 0.06	0.49 ± 0.06
Total protein (g/dL)	6.71 ± 0.36	6.76 ± 0.37	6.21 ± 0.29	6.14 ± 0.26

Mean value ± SD.

**Table 14:** Relative organ weights of rats following oral administration of **C-L-NPs-C-CS-III-M**.

Relative Organ Weight (g/100 g of Body Weight)	Acute Toxicity		Subacute Toxicity	
	Control	C-L-NPs-C-CS-III-M	Control	C-L-NPs-C-CS-III-M
Male rats				
Heart	0.75 ± 0.07	0.79 ± 0.08	0.23 ± 0.02	0.24 ± 0.02
Kidney	1.38 ± 0.18	1.40 ± 0.21	0.41 ± 0.04	0.38 ± 0.03
Liver	10.67 ± 1.01	10.81 ± 1.12	3.18 ± 0.17	3.23 ± 0.19
Spleen	2.41 ± 0.19	2.43 ± 0.20	0.83 ± 0.02	0.87 ± 0.03
Female rats				
Heart	0.55 ± 0.06	0.59 ± 0.07	0.31 ± 0.03	0.30 ± 0.03
Kidney	0.91 ± 0.13	0.92 ± 0.14	0.45 ± 0.04	0.44 ± 0.05
Liver	6.34 ± 0.51	6.37 ± 0.53	3.09 ± 0.27	3.14 ± 0.24
Spleen	1.54 ± 0.16	1.53 ± 0.15	0.68 ± 0.05	0.69 ± 0.06

Mean value ± SD.

## Data Availability

Not applicable.
